# Prognostic value of p53 protein expression for patients with gastric cancer – a multivariate analysis

**DOI:** 10.1038/sj.bjc.6690201

**Published:** 1999-03

**Authors:** Y Maehara, M Tomoda, S Hasuda, A Kabashima, E Tokunaga, Y Kakeji, K Sugimachi

**Affiliations:** Department of Surgery II, Faculty of Medicine, Kyushu University, Fukuoka, Japan

**Keywords:** gastric cancer, p53 expression, prognosis, multivariate analysis

## Abstract

Mutations in the *p53* gene, one of the most common genetic alterations in human cancer, are implicated in tumorigenesis and tumour progression. Although p53 protein expression appears to be correlated to prognosis in patients with malignancy, its prognostic role in gastric cancer has remained controversial. We examined the clinical significance of p53 overexpression in 427 patients with gastric cancer, using multivariate analysis. Tumour sections of gastric cancer tissues from these 427 Japanese patients were stained immunohistochemically with monoclonal antibody PAb1801. The presence of p53 expression was statistically compared with clinicopathological features and post-operative survival, using univariate and multivariate analyses. p53 expression was detected in 38.6% (165 out of 427) of these gastric cancers and immunoreactivity was not observed in normal mucosa adjacent to the tumour. A higher rate of p53 detection was observed among large tumours and in those with a prominent depth of invasion, lymphatic and vascular invasion and lymph node involvement. Prognosis was significantly worse for patients with p53-positive-staining tumours. The 5-year survival rate was 62.5% for patients with p53-negative tumours and 43.3% for those with positive malignancies. p53 expression was a significant prognostic factor for node-positive gastric cancers in subjects undergoing treatment with curative resection, as assessed by Cox regression analysis. Thus, the expression of p53 was closely related to the potential for tumour advance and a poorer post-operative prognosis for patients with gastric cancer. © 1999 Cancer Research Campaign


*British Journal of Cancer* (1999) **79**(7/8), 1255–1261
© 1999 Cancer Research Campaign
Article no. bjoc.1998.0201

### Prognostic value of p53 protein expression for patients with gastric cancer – a multivariate analysis

**Y Maehara, M Tomoda, S Hasuda, A Kabashima, E Tokunaga, Y Kakeji and K Sugimachi**

Department of Surgery II, Faculty of Medicine, Kyushu University, Fukuoka, Japan

**Summary** Mutations in the *p53* gene, one of the most common genetic alterations in human cancer, are implicated in tumorigenesis and tumour progression. Although p53 protein expression appears to be correlated to prognosis in patients with malignancy, its prognostic role in gastric cancer has remained controversial. We examined the clinical significance of p53 overexpression in 427 patients with gastric cancer, using multivariate analysis. Tumour sections of gastric cancer tissues from these 427 Japanese patients were stained immunohistochemically with monoclonal antibody PAb1801. The presence of p53 expression was statistically compared with clinicopathological features and post-operative survival, using univariate and multivariate analyses. p53 expression was detected in 38.6% (165 out of 427) of these gastric cancers and immunoreactivity was not observed in normal mucosa adjacent to the tumour. A higher rate of p53 detection was observed among large tumours and in those with a prominent depth of invasion, lymphatic and vascular invasion and lymph node involvement. Prognosis was significantly worse for patients with p53-positive-staining tumours. The 5-year survival rate was 62.5% for patients with p53-negative tumours and 43.3% for those with positive malignancies. p53 expression was a significant prognostic factor for node-positive gastric cancers in subjects undergoing treatment with curative resection, as assessed by Cox regression analysis. Thus, the expression of p53 was closely related to the potential for tumour advance and a poorer post-operative prognosis for patients with gastric cancer.

**Keywords:** gastric cancer; p53 expression; prognosis; multivariate analysis

The tumour-suppressor gene *p53* is a nuclear protein which binds to and modulates the expression of genes important for DNA repair, cell division and cell death by apoptosis (Harris and Hollstein, 1993; Carson and Lois, 1995). This gene regulates the onset of DNA replication at the G1-S boundary (Vogelstein and Kinzler, 1992), and p53 is also a component of a spindle checkpoint that ensures the maintenance of diploidy (Cross et al, 1995). Abnormalities of the *p53* gene represent the most common genetic alterations in human cancer, and at least half of all tumours have mutations or rearrangements of both copies of the *p53* gene on the short arm of chromosome 17 (Levine et al, 1994; Soussi et al, 1994). It is now well established that most p53 missense mutations alter the conformation of the protein leading to a prolonged half-life. There is an accumulation in the nucleus of the tumour cell, the levels of which can be determined using immunohistochemical methods (Gannon et al, 1990; Levine et al, 1991).

Because wild-type p53 negatively regulates the cell cycle, a loss of function by mutation might be expected to result in enhanced proliferative activity and tumour progression (Chang et al, 1995). There are reports on p53 protein accumulation in breast cancer (Thor et al, 1992; Allred et al, 1993), lung cancer (Quinlan et al, 1992), oesophageal cancer (Shimaya et al, 1993), colon cancer (Yamaguchi et al, 1993), laryngeal cancer (Narayana et al, 1998), nephroblastoma (Govender et al, 1998) and gastric cancer (Martin et al, 1992; Starzynska et al, 1992, Kakeji et al, 1993; Joypaul et al, 1994), and survival time was shortened. These prognostic significances of p53 expression have been investigated from the standpoint of tumour characteristics of higher growth and metastatic potentials (Kakeji et al, 1993; Maehara et al, 1995). However, other studies reported controversial results regarding the clinical value for p53 expression in various cancers (McLaren et al, 1992; Bell et al, 1993; Brambilla et al, 1993; Motojima et al, 1994; Sarbia et al, 1994; Schneider et al, 1994; Gabbert et al, 1995; Piffkò et al, 1998; Suto et al, 1998). Discussions were based on differences in patient populations, immunohistochemical methods, and also the clinical value of p53 protein expression in tumour advance. We investigated the possible clinical role of p53 protein expression in gastric cancer, determined using an immunohistochemical method. We meticulously examined data on 427 patients, using univariate and multivariate analyses.

*Received 19 November 1997*
*Revised 29 May 1998*
*Accepted 2 June 1998*

*Correspondence to:* Y Maehara, Department of Surgery II, Faculty of Medicine, Kyushu University, Fukuoka, 812-8582, Japan

### PATIENTS AND METHODS

### Patients

Between 1974 and 1991, 427 Japanese patients, with primary gastric cancer and no evidence of malignancy in other organs, underwent gastric resection in the Department of Surgery II, Kyushu University Hospital, Fukuoka, Japan. Standardized procedure was that gastric resection was carried out after determining the resection line 3 cm apart from the macroscopic edge for a localized tumour and 6 cm for an infiltrative tumour (Kawasaki, 1975; Bozzetti et al, 1982). Prophylactic lymph node dissection of more than D2 resection was carried out (Maehara et al, 1992*a*). Complete excision of invaded organs was performed, irrespective of the number of sites on the organs, when there was no evidence of incurable factors such as peritoneal dissemination, liver metastasis and widespread nodal involvement (Korenaga et al, 1988). The lymph nodes in groups 1, 2 and 3 are referred to as n1, n2 and n3, respectively, based on lymph node metastasis. Lymph node dissection was classified as follows: D1, complete removal of group 1 lymph node alone; D2, complete removal of group 1 and 2 lymph nodes; and D3, complete removal of group 1, 2 and 3 lymph nodes. The pathological diagnosis and classification was according to the General Rules for the Gastric Cancer Study in Surgery and Pathology in Japan (Japanese Research Society for Gastric Cancer, 1981, 1993). Curability A means no residual tumours with a high probability of cure, under the following conditions: no serosal invasion; n0 treated by D1, 2, 3 or n1 treated by D2 or 3; MO, peritoneal dissemination negative (P0), liver metastasis negative (H0); and the proximal and distal margins > 10 mm. Curability B means no residual tumours but not evaluable as 'curability A', and curability C shows definite residual tumour. Tumour-advance stage was determined by the TNM classification of UICC (Sobin and Wittekind, 1997).

Three patients (0.07%) died within the first 30 post-operative days. Of the remaining 424 patients, 173 are alive at the time of writing this report. Recurrence of the gastric cancer and death occurred in 173 patients, 55 died of another disease and 13 died of undetermined causes. Death owing to causes other than gastric cancer were considered as censored data in the statistical analysis.

### p53 staining

Tissue sections were immunostained with a monoclonal antibody against p53 (PAb1801, Oncogene Science, USA) (Banks et al, 1986; Porter et al, 1992; Sarbia et al, 1994; Oiwa et al, 1995). This antibody recognizes a denaturation-resistant epitope between amino acids 32 and 79 of both wild-type and mutant conformations. Xylene was used to remove paraffin from the 5-µm sections, then the sections were progressively hydrated in decreasing concentrations of ethanol. The slides were placed in a thermoresistant beaker filled with 0.1 M phosphate-buffered saline (PBS) (pH 7.4) and autoclaved at 121°C to allow the fixed embedded tissue antigen to react with the monoclonal antibody. The sections were then cooled down to room temperature for about 20 min and rinsed in PBS. These sections were then covered with normal rabbit serum for 15 min to reduce non-specific staining, then incubated with a 1:100 dilution of primary antibody at room temperature for 1 h. Next, the sections were washed with PBS, incubated with a 1:600 dilution of biotinylated goat anti-mouse IgG (Dako, Denmark) at room temperature for 30 min and then covered with a 1:1000 dilution of labelled streptavidin peroxidase (Dako) at room temperature for 30 min. The antibody was localized with 3,3′-diaminobenzidine tetrahydrochloride and 0.065% sodium azide was used to block endogenous peroxidase.

We stained both the deep periphery of the tumour and adjacent tumour-free tissue. A distinct nuclear immunoreaction for p53 was recorded as positive, and here the nuclear staining pattern was diffuse with little variation. When 10% of the cancer cells showed a positive nuclear staining, a positive-staining was defined (Kakeji et al, 1993).

### Post-operative chemotherapy

All of the patients with advanced stage of gastric cancer (t2–t4) were treated with post-operative chemotherapy (Sugimachi et al, 1997). An intravenous injection of 10 mg mitomycin C (Kyowa Hakko, Japan) was given on the day of operation, and fluorinated pyrimidine UFT (Taiho Pharmaceutical, Japan) orally in a daily dose of 400 mg was started 2 weeks after the operation and was continued for 1 year.

### Statistical analysis

The BMDP Statistical Package program (BMDP; Los Angeles, CA, USA) for the IBM (Armonk, NY, USA) 3090 mainframe computer was used for all analyses (Dixon, 1988). The BMDP P4F and P3S programs were used for the chi-squared test and the Mann–Whitney test to compare data from patients with p53-negative and p53-positive tumours. The BMDP P1L program was used to analyse survival rates by the Kaplan–Meier method, and the Mantel–Cox method to test for equality of the survival curves. The BMDP P2L program was used for simultaneous multivariate adjustment of all covariates by the Cox regression analysis with the forward stepwise model (Cox, 1972). The level of significance was $P < 0.05$.

### RESULTS

The monoclonal antibody pAb1801 has been widely used for p53 protein expression in various cancer tissues. The positive rate of p53 expression in gastric cancer cells was 36.8% (165 out of 427). The staining of p53 was nuclear and p53 was never observed in the normal gastric mucosa examined in all 427 cases, as shown in Figure 1.

### Clinicopathological factors

Table 1 shows clinicopathological data on 262 patients in whom there was no p53 expression and for 165 patients with p53 overexpression. In the p53-positive patients, the entire stomach was more likely to be involved, and the tumour was larger compared with p53-negative cases. There were no differences in tissue differentiation and growth patterns. Serosal invasion was more prominent, lymphatic and vascular involvement was more common and the rate of lymph node metastasis was higher in p53-positive tumours.

Surgical management was also compared between the groups (Table 2). There was no difference with regard to the extent of lymph node dissection. Because the entire stomach was more commonly involved, the rate of total gastrectomy was higher in the p53-positive patients. The rate of operative curability C (non-curative resection) was higher in p53-positive patients because of a more advanced stage of the tumour.

### Survival rates

Post-operative survival curves for patients with p53-negative and p53-positive tumours are shown in Figure 2. The non-gastric cancer deaths were considered as lost to follow-up, as of time of death, and data on survivors with a follow-up time shorter than 10 years were censored from analysis. The 5-year survival rate was 62.5% for p53-negative patients and 43.3% for p53-positive patients ($P < 0.01$).

Next, we determined survival rates for patients with p53-negative and p53-positive tumours for tumour size, serosal invasion,

*© Cancer Research Campaign 1999*

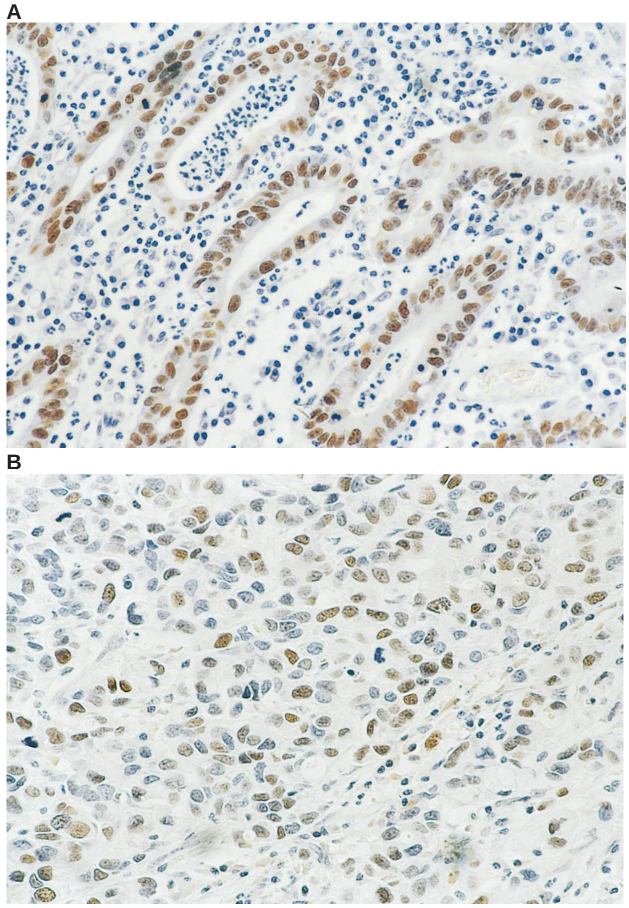

**Figure 1** Immunohistochemical detection of p53 protein in nuclei in paraffin sections using anti-p53 monoclonal antibody PAb1801 (× 180). (**A**) Differentiated gastric carcinoma; (**B**) undifferentiated gastric carcinoma lymphatic and vascular invasion, lymph node metastasis and operative curability, which were identified as factors having a close relation with p53 expression as shown in Tables 1 and 2. In the subgroups of tumour size over 5 cm, serosal invasion positive, lymphatic and vascular invasion positive and lymph node metastasis-positive tumours, and no residual tumours by curative resection (curability A and B), a significantly shorter survival time was noted in p53-positive patients compared with p53-negative patients (Table 3). Survival curves for patients with p53-negative or p53-positive gastric cancers were determined for each node-negative and node-positive group (Figure 3A and B).

### Recurrence pattern in curatively resected patients

Data from patients with p53-positive tumours and p53-negative tumours who underwent curative resection (curability A and B) were analysed for recurrence patterns (Table 4). There was a recurrence in 39 out of 204 cases (19.1%) of p53-negative patients and in 29 out of 106 (27.4%) of p53-positive patients. In both groups, recurrence was noted in the peritoneum, distant organs and lymph nodes.

**Table 1** Clinicopathological characteristics of patients with p53-negative and p53-positive gastric cancer

| Variable | p53 negative ($n$ = 262) | p53 positive ($n$ = 165) | $P$-value |
|---|---|---|---|
| Age (years) | 61.6 ± 12.5[a] | 62.1 ± 12.6 | 0.6871 |
| Sex | | | |
| Men | 175 (66.8) | 120 (72.7) | 0.1964 |
| Women | 87 (33.2) | 45 (27.3) | |
| Tumour maximal diameter (cm) | 5.89 ± 3.87[a] | 7.33 ± 4.19 | 0.0002 |
| Location of tumour | | | |
| Upper | 57 (21.8) | 43 (26.0) | 0.0285 |
| Middle | 84 (32.1) | 35 (21.2) | |
| Lower | 95 (36.2) | 59 (35.8) | |
| Whole stomach | 26 (9.9) | 28 (17.0) | |
| Histology | | | |
| Differentiated | 123 (47.3) | 67 (40.6) | 0.1757 |
| Undifferentiated | 137 (52.7) | 98 (59.4) | |
| Specific[b] | 2 | 0 | |
| Serosal invasion | | | |
| Negative (t1,t2) | 149 (56.9) | 74 (44.8) | 0.0155 |
| Positive (t3,t4) | 113 (43.1) | 91 (55.2) | |
| Histological growth pattern | | | |
| Expansive | 45 (20.9) | 25 (16.7) | 0.4977 |
| Intermediate | 67 (31.2) | 45 (30.0) | |
| Infiltrative | 103 (47.9) | 80 (53.3) | |
| Unknown[b] | 47 | 15 | |
| Lymphatic involvement | | | |
| Negative | 138 (52.9) | 65 (39.6) | 0.0078 |
| Positive | 123 (47.1) | 99 (60.4) | |
| Unknown[b] | 1 | 1 | |
| Vascular involvement | | | |
| Negative | 205 (78.5) | 108 (67.1) | 0.0090 |
| Positive | 56 (21.5) | 53 (32.9) | |
| Unknown[b] | 1 | 4 | |
| Histological lymph node metastasis | | | |
| Negative | 120 (46.5) | 49 (29.9) | 0.0007 |
| Positive | 138 (53.5) | 115 (70.1) | |
| Unknown[b] | 4 | 1 | |
| Peritoneal dissemination | | | |
| Negative | 246 (93.9) | 151 (91.5) | 0.3492 |
| Positive | 16 (6.1) | 14 (8.5) | |
| Liver metastasis | | | |
| Negative | 255 (97.3) | 156 (94.5) | 0.1404 |
| Positive | 7 (2.7) | 9 (5.5) | |
| Stage (UICC) | | | |
| Ia | 80 (30.5) | 28 (17.0) | 0.0089 |
| Ib | 25 (9.5) | 12 (7.3) | |
| II | 39 (14.9) | 29 (17.6) | |
| IIIa | 51 (19.5) | 30 (18.2) | |
| IIIb | 25 (9.5) | 23 (13.9) | |
| IV | 42 (16.1) | 43 (26.0) | |

NS, no significant difference; figures in parentheses are percentages; [a]mean ± standard deviation; [b] unknown and specific cases were excluded from statistical analysis.

### Multivariate analysis of p53 protein expression

Data from Cox regression analysis of all factors are listed in Table 1, and revealed that lymph node metastasis, serosal invasion, liver metastasis, tumour size and peritoneal dissemination proved to be

© Cancer Research Campaign 1999

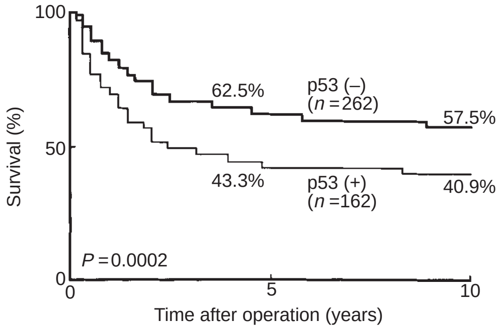

**Figure 2** Survival curves for patients with p53-negative or p53-positive gastric cancers. When the deaths were considered as gastric cancer-related, the patients with p53-positive tumours ($n$ = 165) had a shorter survival time than did those with p53-negative tumours ($n$ = 262) ($P$ = 0.0002). Solid line, p53-negative patients; light line, p53-positive patients

**Table 2** Surgical management of patients with p53-negative or p53-positive gastric cancer

| Variable | p53 negative ($n$ = 262) | p53 positive ($n$ = 165) | $P$-value |
|---|---|---|---|
| Operative procedure | | | |
| Partial | 151 (57.9) | 68 (41.7) | 0.0012 |
| Total | 110 (42.1) | 95 (58.3) | |
| Unknown[a] | 1 | 2 | |
| Lymph node dissection | | | |
| D0 and D1 | 60 (22.9) | 51 (30.9) | 0.0662 |
| D2 and D3 | 202 (77.1) | 114 (69.1) | |
| Curability | | | |
| Curability A, B | 204 (78.5) | 106 (64.2) | 0.0030 |
| Curability C | 56 (21.5) | 59 (35.8) | |

NS, no significant difference; figures in parentheses are percentages; [a]unknown cases were excluded from statistical analysis.

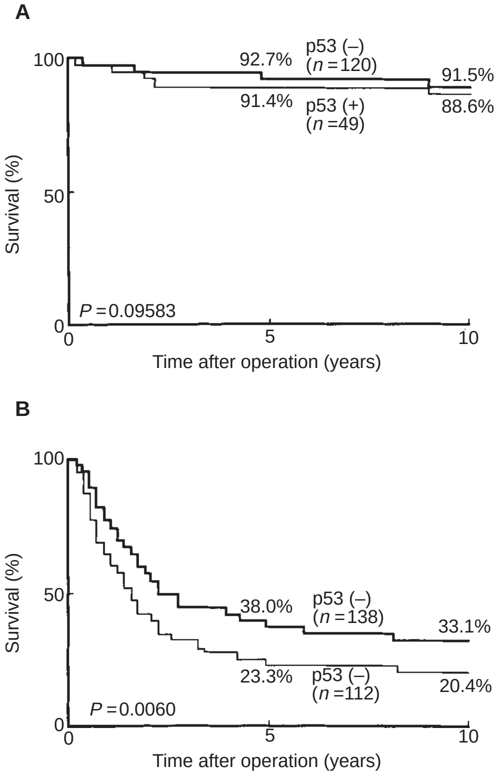

**Figure 3** Survival curves for patients with p53-negative or p53-positive gastric cancers in each node-negative and node-positive group. (**A**) There was no difference between p53-negative ($n$ = 120) and p53-positive patients ($n$ = 49) for the node-negative group ($P$ = 0.9583). (**B**) Survival time for p53-positive patients ($n$ = 115) was shorter than for p53-negative patients ($n$ = 138) in those in the node-positive group ($P$ = 0.0060). Solid line, p53-negative patients; light line, p53-positive patients independent factors related to the prognosis of the patients but not for the p53 expression. Next, we examined the independence of p53 expression in the subgroup of lymph node metastasis-negative and -positive patients treated with curative resection (curability A and B). p53 expression proved to be a significant prognostic factor in patients with node-positive gastric cancer, as shown in Table 5. Factors of age, sex, location of the tumour, histology, histological growth pattern and lymphatic involvement were not significant.

### DISCUSSION

In this study, we made use of immunohistochemical staining to examine the relationship between p53 expression, clinicopathological factors and survival, in tissue samples from 427 patients resected because of gastric cancer.

Alterations in the tumour-suppressor *p53* gene play a role in the genesis of diverse types of human malignancies (Harris and Hollstein, 1993; Soussi et al, 1994; Chang et al, 1995), and allelic losses and point mutations in the counterpart of the locus of the *p53* gene in chromosome 17 are frequent. p53 mutation is a common event in gastric cancer, occurring from the early stage of progression with its specific mutation spectrum (Kim et al, 1991; Yokozaki et al, 1992; Uchino et al, 1993). The mutated *p53* gene loses function as a tumour suppressor and can act as a dominant oncogene (Levine et al, 1991). Loss of p53 function accelerates the process of tumorigenesis and alters the phenotype of cancer cells and the response of cells to agents that damage DNA (Carson and Lois, 1995; Chang et al, 1995). Mutant-type p53 proteins have a prolonged half-life, and are thus more likely than the wild-type protein to be detected using immunohistochemical assays (Finlay et al, 1988; Gannon et al, 1990; Levine et al, 1991).

The antibody PAb1801, which we used, recognizes both the wild-type and mutant forms of p53 (Banks et al, 1986; Porter et al, 1992). No reactivity was observed in any normal gastric mucosa adjacent to the tumour tissue and the normal protein has a very short half-life, suggesting that the immunoreactivity of p53 in the tumour tissue itself is likely to represent mutant forms of p53 and relates to a more malignant biological character. Conversely, 61.4% of the gastric cancers in this study were p53 negative. The tumours with immunohistochemically undetectable p53 could represent a decreased availability of the epitope of p53 protein as a result of fixation in paraffin, tumours with normal levels of wild-type p53, tumours with both alleles of the *p53* gene deleted, or tumours expressing a mutant p53 protein not identified by the antibody used in this study (Gabbert et al, 1995).

© Cancer Research Campaign 1999

**Table 3** Five-year survival rate for patients with p53-negative or-positive gastric cancer in the subgroup for each clinicopathological factor

| | 5-year survival rate (%) | | |
|---|---|---|---|
| **Variable** | **p53 negative** | **p53 positive** | ***P*-value[a]** |
| Tumour size | | | |
| < 5 cm | 84.4 | 78.5 | 0.1228 |
| 5 cm | 42.2 | 23.1 | 0.0155 |
| Serosal invasion | | | |
| Negative | 94.5 | 97.0 | 0.0592 |
| Positive | 42.3 | 28.7 | 0.0027 |
| Lymphatic invasion | | | |
| Negative | 79.4 | 72.5 | 0.2798 |
| Positive | 43.2 | 24.2 | 0.0039 |
| Vascular invasion | | | |
| Negative | 67.4 | 54.2 | 0.0220 |
| Positive | 42.7 | 24.5 | 0.0910 |
| Lymph node metastasis | | | |
| Negative | 92.7 | 91.4 | 0.9580 |
| Positive | 38.0 | 23.3 | 0.0060 |
| Operative curability | | | |
| Curability A, B | 76.3 | 64.5 | 0.0284 |
| Curability C | 9.1 | 6.1 | 0.3404 |

NS, no significant difference; [a]*P*-values for the Mantel–Cox test.

**Table 4** Recurrence after curative resection of curability A and B for patients with p53-negative or p53-positive gastric cancer

| **Recurrence** | **p53 negative (*n* = 204)** | **p53 positive (*n* = 106)** |
|---|---|---|
| Without recurrence | 165 (80.9) | 77 (72.6) |
| With recurrence | 39 (19.1) | 29 (27.4) |
| Peritoneum | 9 | 9 |
| Liver | 12 | 7 |
| Lung | 5 | 5 |
| Bone | 2 | 1 |
| Brain | 3 | 2 |
| Local | 6 | 4 |
| Lymph node | 4 | 3 |
| Unknown | 13 | 8 |

Figures in parentheses are percentages.

**Table 5** Cox regression analysis for patients with node-positive gastric cancer

| **Explanatory variable (observed value)** | ***P*-value** | **Relative risk (95% confidence interval)** |
|---|---|---|
| Tumour size (per cm) | 0.0001 | 1.1147 (1.0693–1.1620) |
| Serosal invasion (none, present) | 0.0107 | 2.1984 (1.4626–3.3042) |
| Venous invasion (none, present) | 0.0402 | 1.4878 (1.0654–2.0777) |
| p53 overexpression (none, present) | 0.0486 | 1.3803 (1.0034–1.8787) |

The p53 abnormal staining was reported to be related to the proliferating activity of cancer cells (Kakeji et al, 1993), and aggressive behaviour of cancer cells for serosal invasion, lymph node metastasis of gastric cancer and a poorer prognosis ensued (Martin et al, 1992; Starzynska et al, 1992; Joypaul et al, 1994; Maehara et al, 1995; Mönig et al, 1997). Because vascular and lymphatic involvement are closely related to p53 expression, p53 expression was closely related to tumour invasion and metastasis and, in particular, proved to be a significant prognostic factor for node-positive cases. In cases of breast cancer, p53 expression was closely related to prognosis for node-negative cancers (Thor et al, 1992; Allred et al, 1993). However, early stages of gastric cancer showed no lymph node metastasis, and the post-operative prognosis was improved in both p53-negative and -positive groups by the surgical treatment (Maehara et al, 1992*b*, 1993).

Other investigators reported that p53 overexpression is not related to the prognosis of human malignacies, including gastric cancer (Motojima et al, 1994; Schneider et al, 1994; Gabbert et al, 1995). McLaren et al (1992) suggested that p53 was of considerable importance in the initiation of tumours in a wide variety of tissues, but the nature of the particular oncogene involved initially is probably of little significance once a tumour has developed. p53 protein expression could also be induced by a number of other factors, i.e. viral infection, oncogene overexpression and transcriptional activation, or mutations outside the conserved regions of p53 (Wynford-Thomas, 1992; Bell et al, 1993). There are reports of a combined assessment of expressions of p53 and of other factors, for example, cyclin E and vascular endothelial growth factor as useful factors for evaluating tumour behaviour and the poor post-operative prognosis for subjects with various cancers (Kang et al, 1997; Furihata et al, 1998; Sakaguchi et al, 1998; Takahashi et al, 1998). Therefore, these approaches may further the clinical usefulness of examining p53 expression.

The present findings show that p53 expression is closely related to tumour invasion and metastasis and a poorer prognosis in humans with gastric cancer. Because p53 is an integral part of anti-cancer-related DNA damage and apoptotic pathways (Caelles et al, 1994; Carson and Lois, 1995), the clonal expansion of cells that acquire mutations in the *p53* gene reveals resistance to cancer chemotherapy of solid tumours (Lowe et al, 1994). Post-operative chemotherapy proved to be non-effective for p53-negative breast cancer, therefore accumulation of p53 protein can lead to enhanced chemoresistance (Koechli et al, 1994; Elledge et al, 1995). Chin et al (1992) reported that the multidrug resistance gene could be activated during the tumour progression associated with mutations in p53. Almost all of our patients with advanced gastric cancer were prescribed post-operative chemotherapy, therefore the relation between p53 protein expression and the effect of post-operative chemotherapy could not be determined. An appropriate treatment for patients with gastric cancer with p53 expression has yet to be determined.

*© Cancer Research Campaign 1999* 

### ACKNOWLEDGEMENTS

This work was supported by a Grant-in-Aid for Scientific Research from the Ministry of Education, Science, Sports and Culture of Japan and a public trust fund for the promotion of surgery. We thank M Ohara for comments and H Baba, Y Matsuo and J Tsuchihashi for technical assistance.

### REFERENCES

Allred DC, Clark GM, Elledge R, Fuqua SAW, Brown RW, Chamness GC, Osborne CK and McGuire WL (1993) Association of p53 protein expression with tumor cell proliferation rate and clinical outcome in node-negative breast cancer. *J Natl Cancer Inst* **85**: 200–206

Banks L, Matlashewski M and Crawford L (1986) Isolation of human-p53-specific monoclonal antibodies and their use in the studies of human p53 expression. *Eur J Biochem* **159**: 529–534

Bell SM, Scott N, Cross D, Sagar P, Lewis FA, Blair GE, Taylor GR, Dixon MF and Quirke P (1993) Prognostic value of p53 overexpression and c-Ki-*ras* gene mutations in colorectal cancer. *Gastroenterology* **104**: 57–64

Bozzetti F, Bonfanti G, Bufalino R, Menotti V, Persann S, Andreola S, Doci R and Gennari L (1982) Adequacy of margins of resection in gastrectomy for cancer. *Ann Surg* **196**: 685–690

Brambilla E, Gazzeri S, Moro D, de Fromentel CC, Gouyer V, Jacrot M and Brambilla C (1993) Immunohistochemical study of p53 in human lung carcinomas. *Am J Pathol* **143**: 199–210

Caelles C, Helmberg A and Karin M (1994) p53-dependent apoptosis in the absence of transcriptional activation of p53-target genes. *Nature* **370**: 220–223

Carson DA and Lois A (1995) Cancer progression and p53. *Lancet* **346**: 1009–1011

Chang F, Syrjänen S and Syrjänen K (1995) Implications of the p53 tumor-suppressor gene in clinical oncology. *J Clin Oncol* **13**: 1009–1022

Chin K-V, Ueda K, Pastan I and Gottesman MH (1992) Modulation of activity of the promoter of the human MDR1 gene by ras and p53. *Science* **255**: 459–462

Cox DR (1972) Regression models and life tables. *J R Stat Soc Series B* **34**: 187–220

Cross SM, Sanchez CA, Morgan CA, Schimke MK, Ramel S, Idzerda RL, Raskind WH and Reid BJA (1995) A p53-dependent mouse spindle checkpoint. *Science* **267**: 1353–1356

Dixon WJ (1988) *BMDP Statistical Software*. University of California Press: Berkeley, CA

Elledge RM, Gray R, Mansour E, Yu Y, Clark GM, Ravdin P, Osborne CK, Gilchrist K, Davidson NE, Robert N, Tormey DC and Allred DC (1995) Accumulation of p53 protein as a possible predictor of response to adjuvant combination chemotherapy with cyclophosphamide, methotrexate, fluorouracil, and prednisone for breast cancer. *J Natl Cancer Inst* **87**: 1254–1256

Finlay CA, Hinds PW, Tan T-H, Eliyahu D, Oren M and Levine AJ (1988) Activating mutations for transformation by p53 produce a gene product that forms an hsc 70–p53 complex with an altered half-life. *Mol Cell Biol* **8**: 531–539

Furihata M, Ohtsuki Y, Sonobe H, Shuin T, Yamamoto A, Terao N and Kuwahara M (1988) Prognostic significance of cyclin E and p53 protein overexpression in carcinoma of the renal pelvic and ureter. *Br J Cancer* **77**: 783–788

Gabbert HE, Müller W, Schneiders A, Meier S and Hommel G (1995) The relationship of p53 expression to the prognosis of 418 patients with gastric carcinoma. *Cancer* **76**: 720–726

Gannon JV, Greaves R, Iggo R and Lane DP (1990) Activating mutations in p53 produce a common conformational effect. A monoclonal antibody specific for the mutant form. *EMBO J* **9**: 1595–1602

Govender D, Harilal P, Hadley GP and Chetty R (1998) p53 protein expression in nephroblastomas: a predictor of poor prognosis. *Br J Cancer* **77**: 314–318

Harris CC and Hollstein M (1993) Clinical implications of the p53 tumor-suppressor gene. *N Engl J Med* **329**: 1318–1327

Japanese Research Society for Gastric Cancer (1981) The general rules for the gastric cancer study in surgery and pathology. Part I. Clinical classification. *Jpn J Surg* **11**: 127–139. Part II. Histological classification of gastric cancer. *Jpn J Surg* **11**: 140–145

Japanese Research Society for Gastric Cancer (ed) (1993) *The General Rules for Gastric Cancer Study* (in Japanese), 12th edn. Kanehara and Co.: Tokyo, Japan

Joypaul BV, Hopwood D, Newman EL, Qureshi S, Grant A, Ogston SA, Lane DP and Cuschieri A (1994) The prognostic significance of the accumulation of p53 tumour-suppressor gene protein in gastric adenocarcinoma. *Br J Cancer* **69**: 943–946

Kakeji Y, Korenaga D, Tsujitani S, Baba H, Anai H, Maehara Y and Sugimachi K (1993) Gastric cancer with p53 overexpression has high potential for metastasising to lymph nodes. *Br J Cancer* **67**: 589–593

Kang S-M, Maeda K, Onoda N, Chung Y-S, Nakata B, Nishiguchi Y and Sowa M (1997) Combined analysis of p53 and vascular endothelial growth factor expression in colorectal carcinoma for determination of tumor vascularity and liver metastasis. *Int J Cancer* **74**: 502–507

Kawasaki S (1975) A clinicopathological study on upward intramural extension of cancer of the stomach (in Japanese with English abstract). *Fukuoka Acta Med* **66**: 1–23

Kim J-H, Takahashi T, Chiba I, Park J-G, Birrer MJ, Roh JK, Lee HD, Kim J-P, Minna JD and Gazdar AF (1991) Occurrence of p53 gene abnormalities in gastric carcinoma tumors and cell lines. *J Natl Cancer Inst* **83**: 938–943

Koechli OR, Schaer GN, Seifert B, Hornung R, Haller U, Eppenberger U and Mueller H (1994) Mutant p53 protein associated with chemosensitivity in breast cancer specimens. *Lancet* **344**: 1647–1648

Korenaga D, Okamura T, Baba H, Saito A and Sugimachi K (1988) Results of resection of gastric cancer extending to adjacent organs. *Br J Surg* **75**: 12–15

Levine AJ, Momand J and Finlay CA (1991) The p53 tumour suppressor gene. *Nature* **351**: 453–456

Levine AJ, Perry ME, Chang A, Silver A, Dittmer D, Wu M and Welsh D (1994) The 1993 Walter Hubert Lecture: The role of the p53 tumour-suppressor gene in tumorigenesis. *Br J Cancer* **69**: 409–416

Lowe SW, Bodis S, McClatchey A, Remington L, Ruley HE, Fisher DE, Housman DE and Jacks T (1994) p53 status and the efficacy of cancer therapy in vivo. *Science* **266**: 807–810

Maehara Y, Okuyama T, Moriguchi S, Orita H, Kusumoto H, Korenaga D and Sugimachi K (1992*a*) Prophylactic lymph node dissection in patients with advanced gastric cancer promotes increased survival time. *Cancer* **70**: 392–395

Maehara Y, Orita H, Okuyama T, Moriguchi S, Tsujitani S, Korenaga D and Sugimachi K (1992*b*) Predictors of lymph node metastasis in early gastric cancer. *Br J Surg* **79**: 245–247

Maehara Y, Okuyama T, Oshiro T, Baba H, Anai H, Akazawa K and Sugimachi K (1993) Early carcinoma of the stomach. *Surg Gynecol Obstet* **177**: 593–597

Maehara Y, Okuyama T, Kakeji Y, Endo K, Yamamoto M and Sugimachi K (1995) A tumour-associated cell-surface glycoprotein accompanying p53 overexpression and higher growth potential for gastric cancer. *Br J Cancer* **71**: 999–1002

Martin HM, Filipe MI, Morris RW, Lane DP and Silvestre F (1992) p53 expression and prognosis in gastric carcinoma. *Int J Cancer* **50**: 859–862

McLaren R, Kuzu I, Dunnill M, Harris A, Lane D and Gatter KC (1992) The relationship of p53 immunostaining to survival in carcinoma of the lung. *Br J Cancer* **66**: 735–738

Mönig SP, Eidt S, Zirbes TK, Stippel D, Baldus SE and Pichlmaier H (1997) p53 expression in gastric cancer. Clinicopathological correlation and prognostic significance. *Dig Dis Sci* **42**: 2463–2467

Motojima K, Furui J, Kohara N, Ito T and Kanematsu T (1994) Expression of p53 protein in gastric carcinomas is not independently prognostic. *Surgery* **116**: 890–895

Narayana A, Vaughan ATM, Gunaratne S, Kathuria S, Walter SA and Reddy SP (1998) Is p53 an independent prognostic factor in patients with laryngeal carcinoma? *Cancer* **82**: 286–291

Oiwa H, Maehara Y, Ohno S, Sakaguchi Y, Ichiyoshi Y and Sugimachi K (1995) Growth pattern and p53 overexpression in patients with early gastric cancer. *Cancer* **75**: 1454–1459

Piffkò J, Bànkfalvi À, Tory K, Füzesi L, Bryne M, Öfner D, Kusch F, Joos U and Schmid KW (1998) Molecular assessment of p53 abnormalities at the invasive front of oral squamous cell carcinomas. *Head Neck* **20**: 8–15

Porter PL, Gown AM, Kramp SG and Coltrerea MD (1992) Widespread p53 overexpression in human malignant tumors. *Am J Pathol* **140**: 145–153

Quinlan DC, Davidson AG, Summers CL, Warden HE and Doshi HM (1992) Accumulation of p53 protein correlates with a poor prognosis in human lung cancer. *Cancer Res* **52**: 4828–4831

Sakaguchi T, Watanabe A, Sawada H, Yamada Y, Yamashita J, Matsuda M, Nakajima M, Miwa T, Hirao T and Nakano H (1998) Prognostic value of cyclin E and p53 expression in gastric carcinoma. *Cancer* **82**: 1238–1243

Sarbia M, Porschen R, Borchard F, Horstmann O, Willers R and Gabbert HE (1994) p53 protein expression and prognosis in squamous cell carcinoma of the esophagus. *Cancer* **74**: 2218–2223

Schneider BG, Hilsenbeck SG, Hensel CH, Pekkel V, Shelton CH, Rodríguez-Martínez HA, Gutiérrez-Díaz CME, Pulitzer DR and Allred DC (1994) p53 mutations in gastric and colorectal cancers in Texas Hispanics versus Anglos. *Virchows Arch* **424**: 187–193

 *© Cancer Research Campaign 1999*

Shimaya K, Shiozaki H, Inoue M, Tahara H, Monden T, Shimano T and Mori T (1993) Significance of p53 expression as prognostic factor in oesophageal squamous cell carcinoma. *Virchows Arch A Pathol Anat* **422**: 271–276

Sobin LH and Wittekind C (1997) *TNM Classification of Malignant Tumour*, 5th edn. John Wiley & Sons: New York, NY

Soussi T, Legros Y, Lubin R, Ory K and Schlichtholz B (1994) Multifactorial analysis of p53 alteration in human cancer: a review. *Int J Cancer* **57**: 1–9

Starzynska T, Bromley M, Ghosh A and Stern PL (1992) Prognostic significance of p53 overexpression in gastric and colorectal carcinoma. *Br J Cancer* **66**: 558–562

Sugimachi K, Maehara Y, Ogawa M, Kakegawa T and Tomita, M (1997) Dose intensity of uracil and tegafur in postoperative chemotherapy for patients with poorly differentiated gastric cancer. *Cancer Chemother Pharmacol* **40**: 233–238

Suto T, Sugai T, Nakamura S, Funato O, Nitta H, Sasaki R, Kanno S and Saito K (1998) Assessment of the expression of p53, MIB-1 (Ki-67 antigen), and argyrophilic nucleolar organizer regions in carcinoma of the extrahepatic bile duct. *Cancer* **82**: 86–95

Takahashi Y, Bucana CD, Cleary KR and Ellis LM (1998) p53, vessel count, and vascular endothelial growth factor expression in human colon cancer. *Int J Cancer* **79**: 34–38

Thor AD, Moore II DH, Edgerton SM, Kawasaki ES, Reihsaus E, Lynch HT, Marcus JN, Schwartz L, Chen L-C, Mayall BH and Smith HS (1992) Accumulation of p53 tumor suppressor gene protein: an independent marker of prognosis in breast cancers. *J Natl Cancer Inst* **84**: 845–855

Uchino S, Noguchi M, Ochiai A, Saito T, Kobayashi M and Hirohashi S (1993) p53 mutation in gastric cancer: a genetic model for carcinogenesis is common to gastric and colorectal cancer. *Int J Cancer* **54**: 759–764

Vogelstein B and Kinzler KW (1992) p53 function and dysfunction. *Cell* **70**: 523–526

Wynford-Thomas D (1992) p53 in tumour pathology: can we trust immunocytochemistry? *J Pathol* **166**: 329–330

Yamaguchi A, Nakagawara G, Kurosaka Y, Nishimura G, Yonemura Y and Miyazaki I (1993) p53 immunoreaction in endoscopic biopsy specimens of colorectal cancer, and its prognostic significance. *Br J Cancer* **68**: 399–402

Yokozaki H, Kuniyasu H, Kitadai Y, Nishimura K, Todo H, Ayhan A, Yasui W, Ito H and Tahara E (1992) p53 point mutations in primary gastric carcinomas. *J Cancer Res Clin Oncol* **119**: 67–70

*© Cancer Research Campaign 1999*
